# Beneficial effects of luseogliflozin on lipid profile and liver function in patients with type 2 diabetes mellitus (BLUE trial): a single-center, single-arm, open-label prospective study

**DOI:** 10.1186/s13098-023-01074-1

**Published:** 2023-05-11

**Authors:** Yuriko Hajika, Yuji Kawaguchi, Kenji Hamazaki, Yasuro Kumeda

**Affiliations:** Department of Internal Medicine, Minami Osaka Hospital, 1-18-18 Higashikagaya, Suminoe-Ku, Osaka, 559-0012 Japan

**Keywords:** Lipids, Non-alcoholic fatty liver disease, Sodium-glucose transporter 2 inhibitor

## Abstract

**Background:**

Arteriosclerosis and non-alcoholic fatty liver disease are major complications of diabetes mellitus. Hyperglycemia, insulin resistance, obesity, and metabolic syndrome are associated with the progression of these complications. Sodium-glucose transporter 2 inhibitors such as luseogliflozin are oral hypoglycemic agents that reduce glucose levels, induce loss of weight or body fat, and improve liver function. However, the effects of these agents on lipid profiles are unclear. Therefore, this study aimed to investigate these effects and their relationship with arteriosclerosis and non-alcoholic fatty liver disease.

**Methods:**

This single-center, single-arm, open-labeled prospective study enrolled 25 outpatients with type 2 diabetes mellitus who visited Minami Osaka Hospital. Laboratory tests and body measurements were performed at weeks 0 and 24. Luseogliflozin was started at 2.5 mg/day after breakfast, and data from weeks 0 and 24 were evaluated. There were no changes in the doses of other antidiabetic and dyslipidemia drugs a month prior to or during the study.

**Results:**

The patients showed significant reductions in the levels of triglycerides, remnant-like particle cholesterol, and triglyceride/high-density lipoprotein cholesterol ratio, along with significant increases in the levels of high-density lipoprotein cholesterol and apolipoprotein A-1. Alanine aminotransferase, γ-glutamyl transpeptidase, and the fatty liver index were significantly reduced.

**Conclusions:**

Luseogliflozin-induced changes in the lipid profile were related to the suppression or improvement of arteriosclerosis and liver function, respectively. Patients who received this drug also showed improvements in the levels of liver enzymes and reductions in the fatty liver index. Earlier use of luseogliflozin might prevent diabetic complications.

*Trial registration* This study was registered in the University Hospital Medical Information Network Clinical Trial Registry (UMIN 000043595) on April 6th, 2021

**Supplementary Information:**

The online version contains supplementary material available at 10.1186/s13098-023-01074-1.

## Background

Arteriosclerotic diseases such as cardiovascular disease, cerebrovascular disease, and peripheral arterial disease are some of the complications of diabetes. Arteriosclerotic diseases were the third leading cause of death in patients with diabetes in Japan from 2001 to 2010, following malignant neoplasms and infectious diseases [1]. Other complications associated with diabetes include non-alcoholic fatty liver disease (NAFLD), which can progress to steatohepatitis, cirrhosis, or liver cancer without treatment. NAFLD is characterized by triglyceride (TG) accumulation in the liver that is not caused by drugs or alcohol. The development of this disease is associated with hyperglycemia, insulin resistance, obesity, and metabolic syndrome [2]. In patients with diabetes, these risk factors influence each other; therefore, comprehensive control is important for preventing the onset and progression of complications.

Sodium-glucose transporter 2 inhibitors (SGLT2-i) restrict the reabsorption of glucose in the proximal tubule of the kidney and increases the urinary excretion of glucose [3]. In addition to glycemic control, these drugs can induce loss of body weight and visceral fat mass and improve liver function. Some clinical studies have also shown reductions in TG levels and increased high-density lipoprotein (HDL)-cholesterol (C) levels in patients treated with these drugs. However, the effects on low-density lipoprotein (LDL)-C levels vary [4]. Gürkan et al. reported that 6 months of dapagliflozin therapy decreased the serum levels of LDL-C and TGs in patients with type 2 diabetes mellitus (T2DM) [5], while Cha et al. found that SGLT2-i therapy for 24 weeks significantly increased HDL-C and LDL-C levels in patients with T2DM [6]. In a study on empagliflozin, LDL-C and apolipoprotein (Apo) B levels increased [7], but in another study, canagliflozin administration significantly reduced serum TG and total cholesterol levels but showed no obvious effect on HDL-C and LDL-C levels [8]. Luseogliflozin, an SGLT2-i developed by Taisho Pharmaceutical Co., Ltd. in Japan, has been shown to reduce waist circumference, body fat, and TG levels, as well as increase HDL-C levels [9]. Luseogliflozin was also reported to be well tolerated in individuals with normal hepatic function or those with mild or moderate hepatic function [10], probably because luseogliflozin is metabolized by several enzymes in the liver [11], whereas other SGLT2-i are not. Since luseogliflozin differs from other SGLT2-i with respect to its tolerability, we chose this drug to evaluate its potential effects on arteriosclerosis and liver function. To the best of our knowledge, the precise effects of luseogliflozin on the lipid profile remain unclear, especially regarding its effect on ApoA-1, ApoB, remnant-like lipoprotein (RLP)-C, and the TG/HDL-C ratio. Lipids are reported to be associated with arteriosclerosis and fatty liver. Although arteriosclerosis and fatty liver are different conditions, changes in lipids are considered to be the basis of changes in these diseases.

Therefore, we investigated the changes in lipid profile, while using luseogliflozin, by collecting data on both arteriosclerosis and NAFLD. By determining the changes in glucose levels and lipid profile, we aimed to evaluate the potential effects of luseogliflozin on arteriosclerosis and liver function.

## Methods

### Participants

Twenty-five patients with T2DM who visited Minami Osaka Hospital as outpatients were enrolled in this study (Additional file 1). The details of the inclusion and exclusion criteria are provided in Additional file 2. Patients who had a change in their dose or usage of antidiabetic or dyslipidemia drugs within the previous month were excluded. Patients who were 20 years or older, with glycated hemoglobin (HbA1c) values between 7 and 10% (despite being on diet, exercising, and receiving glucose-lowering therapy for at least a month) and fasting plasma TG levels between 120 and 400 mg/dL, were eligible for this study. In accordance with the 1964 Declaration of Helsinki, the purpose of this study was explained to the patients, and written informed consent was obtained.

### Study design

**Fig. 1:**
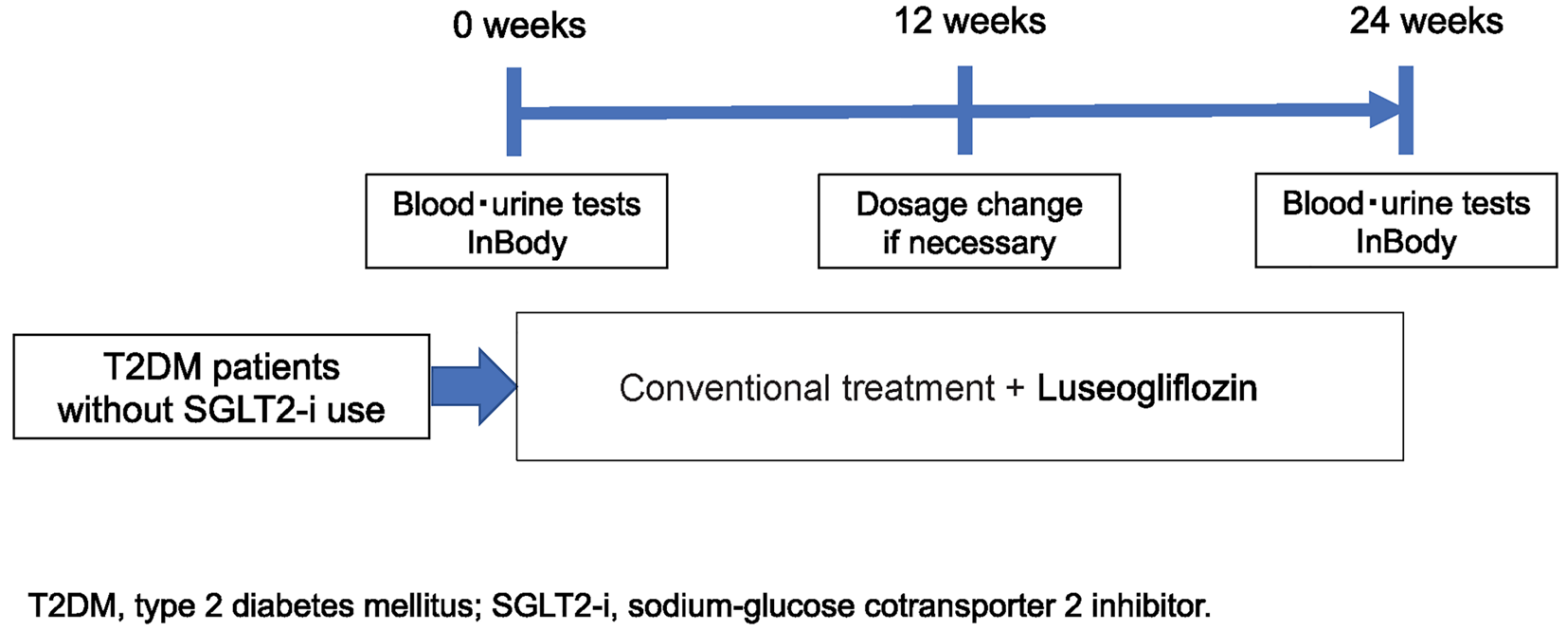
Study design

This single-center, single-arm, open-label, prospective study was conducted with the approval of the institutional review board at Minami Osaka Hospital (No. 2021-2). This study was registered in the University Hospital Medical Information Network Clinical Trial Registry (UMIN 000043595). The study design is illustrated in Fig. 1. The dosage of drugs for diabetes or dyslipidemia was not changed during the study. Laboratory tests (blood and urine tests) and InBody measurements were performed at 0 and 24 weeks after starting the study. InBody measurements were obtained using a Body Composition Analyzer (InBody S10; InBody Japan Inc., Tokyo, Japan). Treatment with luseogliflozin was started at 2.5 mg/day after breakfast, and the dose was raised to 5 mg/day if glycemic control was insufficient at 12 weeks. Data from weeks 0 and 24 were evaluated.

### Laboratory and clinical parameters

Laboratory test samples were collected as fasting specimens. Each lipid type was measured by using the following kits; TG: Cholestest TG, SEKISUI MEDICAL CO., LTD., Tokyo, Japan (Accuracy: 90–110% of the expected assay value, Within-run Reproducibility: Coefficient of variation ≤ 3%), Total cholesterol: Cholestest CHO, SEKISUI MEDICAL CO., LTD., Tokyo, Japan (Accuracy: 95–105% of the expected assay value, Within-run Reproducibility: Coefficient of variation ≤ 3%), HDL-C: Cholestest N HDL, SEKISUI MEDICAL CO., LTD., Tokyo, Japan (Accuracy: 90–110% of the expected assay value, Within-run Reproducibility: Coefficient of variation ≤ 5%), LDL-C: Cholestest LDL, SEKISUI MEDICAL CO., LTD., Tokyo, Japan (Accuracy: 90–110% of the expected assay value, Within-run Reproducibility: Coefficient of variation ≤ 5%). LDL-C was measured by the direct method, as it is not affected by the number of TG-rich lipoproteins. ApoA-1: Apo A-I Auto・N”DAIICHI”, SEKISUI MEDICAL CO., LTD., Tokyo, Japan (Accuracy: 90–110% of the expected assay value, Within-run Reproducibility: Coefficient of variation ≤ 5%), ApoB: ApoB Auto・N”DAIICHI”, SEKISUI MEDICAL CO., LTD., Tokyo, Japan (Accuracy: 90–110% of the expected assay value, Within-run Reproducibility: Coefficient of variation ≤ 5%), RLP-C: MetaboRead RemL-C, Minaris Medical Co., Ltd., Tokyo, Japan (Accuracy: 90–110% of the expected assay value, Within-run Reproducibility: Coefficient of variation ≤ 5%), and RLP-C levels were measured using a homogeneous assay.

For the assessment of NAFLD, the fatty liver index (FLI) was used as a simple predictor of the probability of having a fatty liver. While the most robust approach for diagnosing NAFLD comprises the evaluation of TG accumulation via magnetic resonance imaging or vibration‐controlled transient elastography, as well as a liver biopsy to accurately quantify fibrosis, these methods are not yet applicable in routine practice. Therefore, we chose non-invasive methods for this study. The FLI was calculated from four items: TG level, body mass index (BMI), γ-glutamyl transpeptidase (γ-GTP), and waist circumference. The FLI was calculated as follows: FLI = (e^0.953* log^_e_^(TG) + 0.139*BMI + 0.718* log^_e_^(γ−GTP) + 0.053*waist circumference – 15.745)^/(1 + e^0.953* log^_e_^(TG) + 0.139*BMI + 0.718* log^_e_^(γ−GTP) + 0.053*waist circumference – 15.745^) × 100. A previous study showed that an FLI < 30 indicated that the probability of having fatty liver was 12.3%; an FLI ≥ 30 and ≤ 60 indicated a probability of 58%, and an FLI > 60 indicated a probability of 87% [12].

The fibrosis-4 (FIB-4) index can help determine the underlying fibrosis associated with NAFLD. A FIB-4 index < 1.3 indicates the absence of advanced fibrosis, while a FIB-4 index > 2.67 indicates the presence of advanced fibrosis [13]. The FIB-4 index was calculated based on age, aspartate aminotransferase (AST) and alanine aminotransferase (ALT) levels, and platelet count. The calculation formula was FIB-4 index = age (years) × $\frac{\mathrm{AST}}{\mathrm{platelets}}$ × $\sqrt{\mathrm{ALT}}$ [14].

### Study endpoints

The primary endpoints were changes in HbA1c and lipid metabolism markers, including total cholesterol, TG, HDL-C, LDL-C, non-HDL-C, ApoA-1, ApoB, RLP-C, and TG/HDL-C ratio at 0 and 24 weeks. Secondary endpoints were changes in blood pressure, body weight, BMI, waist circumference (measured at the height of the navel), body fat percentage, skeletal muscle mass, fasting plasma glucose level, serum C-peptide, AST, ALT, γ-GTP, type IV collagen, FIB-4 index, FLI, urinary albumin-creatinine ratio (UACR), estimated glomerular filtration rate (eGFR), red blood cell count, hematocrit, and platelet count at 0 and 24 weeks.

### Statistical analyses

Statistical analyses were performed using R version 4.1.2 (R Foundation for Statistical Computing, Vienna, Austria). The primary and secondary endpoints were evaluated using a paired t-test; items that showed a non-normal distribution were evaluated using the Wilcoxon signed-rank test. Data were presented as mean ± standard deviation, or median (interquartile range). Correlations were calculated using Pearson’s test; items that showed a non-normal distribution were evaluated using Spearman’s test. Statistical significance was set at p < 0.05. For multivariate analysis to investigate the main cause of the changes in FLI or ALT levels, a stepwise variable selection method was used with both entry and retention criteria set at p = 0.2.

## Results

### Patient characteristics

Patient characteristics are shown in Table 1. Twenty-five patients (16 men and 9 women) with T2DM who were visiting our hospital were enrolled in this study. The baseline values for the patient characteristics were as follows: age, 69.8 ± 11.3 years; duration of diabetes, 7.8 ± 5.4 years; HbA1c level, 7.8 ± 0.6%; fasting plasma glucose level, 175.5 ± 37.2 mg/dL; and fasting TG level, 175.0 [147.0–219.0] mg/dL. No adverse events, such as subjective hypoglycemia, urinary tract infection, genital infection, cutaneous symptoms, ketosis, or lower limb necrosis, occurred during the study period [15]. All participants completed the study without dropping out. The dose was raised to 5 mg/day at 12 weeks in 12 of 25 patients.

**Table 1:** Patient characteristics (n = 25)

	Mean ± SD Median (interquartile range)
Age (years)	69.8 ± 11.3
Duration of diabetes (years)	7.8 ± 5.4
Sex (male/female)	16/9
HbA1c (%)	7.8 ± 0.6
FPG (mg/dL)	175.5 ± 37.2
Body weight (kg)	74.2 ± 19.2
BMI (kg/m^2^)	28.4 ± 4.9
Waist circumference (cm)	99.2 ± 11.6
TG (mg/dL)	175.0 [147.0–219.0]
HDL-C (mg/dL)	47.4 ± 7.1
LDL-C (mg/dL)	91.9 ± 30.4
non-HDL-C (mg/dL)	125.4 ± 31.4
FLI	68.0 ± 22.1
Antihyperglycemic drugs	Number (%)
Metformin	16 (64)
DPP-4 inhibitor	21 (84)
Sulphonylurea	6 (24)
Glinide	2 (8)
α-Glucosidase inhibitor	5 (20)
Thiazolidinediones	1 (4)
Insulin	2 (8)
GLP-1 receptor agonist	1 (4)
Antilipidemic drugs	
Statins	15 (60)
Fibrates	1 (4)
Intestinal cholesterol absorption inhibitor	2 (8)
Omega-3 fatty acids	3 (12)

Data are presented as the mean ± SD or median (interquartile range)

HbA1c: glycated hemoglobin; FPG: fasting plasma glucose; BMI: body mass index; TG: triglyceride; HDL-C: high-density lipoprotein-cholesterol; LDL-C: low-density lipoprotein-cholesterol; FLI: fatty liver index; DPP-4: dipeptidyl peptidase-4; GLP-1: glucagon-like peptide-1; SD: standard deviation

### Study endpoints at 24 weeks

Table 2 shows the findings of the primary endpoints at 24 weeks. The HbA1c level decreased significantly (− 0.6 ± 0.9%, p = 0.003) at 24 weeks. As for lipids, HDL-C (+ 5.2 ± 6.7 mg/dL, p < 0.001) and ApoA-1 (+ 7.9 ± 15.5 mg/dL, p = 0.018) levels increased significantly. On the other hand, significant reductions were observed in TG (− 30.0 [− 75.0 to − 10.0] mg/dL, p = 0.007) and RLP-C (− 1.8 ± 3.2 mg/dL, p = 0.010) levels, as well as the TG/HDL-C ratio (− 1.0 [− 1.9 to − 0.4], p = 0.024). No significant changes were observed in total cholesterol, non-HDL-C, LDL-C, or ApoB levels.

**Table 2:** Primary study endpoints at 24 weeks (n = 25)

	0 weeks	24 weeks	Amount of change	p-value
HbA1c (%)	7.8 ± 0.6	7.2 ± 0.8	− 0.6 ± 0.9	0.003*
TG (mg/dL)	175.0 [147.0–219.0]	138.0 [93.0–172.0]	− 30.0 [− 75.0 to − 10.0]	0.007*
Total cholesterol (mg/dL)	172.8 ± 32.0	175.1 ± 37.6	2.3 ± 19.3	0.554
HDL-C (mg/dL)	47.4 ± 7.1	52.5 ± 9.5	5.2 ± 6.7	< 0.001*
LDL-C (mg/dL)	91.9 ± 30.4	93.5 ± 34.0	1.6 ± 14.4	0.584
non-HDL-C (mg/dL)	125.4 ± 31.4	122.6 ± 35.1	− 2.8 ± 19.0	0.468
ApoB (mg/dL)	82.4 ± 19.6	82.6 ± 22.6	0.2 ± 15.6	0.960
ApoA-1 (mg/dL)	146.4 ± 19.8	154.2 ± 25.8	7.9 ± 15.5	0.018*
RLP-C (mg/dL)	8.1 ± 3.0	6.3 ± 3.6	− 1.8 ± 3.2	0.010*
TG/HDL-C ratio	3.9 [2.8–4.6]	2.6 [1.7–3.4]	− 1.0 [− 1.9 to − 0.4]	0.024*

Data are presented as the mean ± standard deviation or median (interquartile range)

*Indicates statistical significance. Paired t-test, (versus 0 weeks) *p < 0.05; Wilcoxon signed-rank test, (versus 0 weeks) *p < 0.05

“Amount of change” is the change between 0 and 24 weeks

HbA1c: glycated hemoglobin; TG: triglyceride; HDL-C: high-density lipoprotein-cholesterol; LDL-C: low-density lipoprotein-cholesterol; Apo: apolipoprotein; RLP-C: remnant-like lipoprotein cholesterol

Table 3 shows the findings for the secondary endpoints at 24 weeks. Systolic blood pressure (− 10.3 ± 14.4 mmHg, p = 0.002), body weight (− 2.2 ± 3.8 kg, p = 0.007), BMI (− 0.8 ± 1.2 kg/m^2^, p = 0.002), and waist circumference (− 2.7 ± 3.6 cm, p < 0.001) were all significantly reduced. Significant reductions were also observed in the fasting plasma glucose level (− 30.4 ± 43.3 mg/dL, p = 0.002), serum C-peptide level (− 0.9 ± 2.16 ng/mL, p = 0.044), ALT level (− 5.6 ± 8.8 IU/L, p = 0.004), γ-GTP level (− 11.0 [− 25.0 to − 1.0] IU/L, p < 0.001), and FLI (− 14.6 ± 15.7, p < 0.001). Significant increases were observed in the red blood cell count (+ 31.0 ± 15.6 × 10^4^/μL, p < 0.001) and hematocrit (+ 3.1 ± 1.8%, p < 0.001). No significant changes were observed in the diastolic blood pressure, body fat percentage, skeletal muscle mass, AST level, type IV collagen level, FIB-4 index, platelet count, and eGFR. Although the UACR showed a decreasing trend, it did not reach statistical significance.

**Table 3:** Secondary study endpoints at 24 weeks (n = 25)

	0 weeks	24 weeks	Amount of change	p-value
FPG (mg/dL)	175.5 ± 37.2	145.0 ± 23.1	− 30.4 ± 43.3	0.002*
Body weight (kg)	74.2 ± 19.2	72.0 ± 19.2	− 2.2 ± 3.8	0.007*
BMI (kg/m^2^)	28.4 ± 4.9	27.6 ± 5.3	− 0.8 ± 1.2	0.002*
Waist circumference (cm)	99.2 ± 11.6	96.4 ± 12.3	− 2.7 ± 3.6	< 0.001*
Body fat percentage (%)	32.2 ± 7.4	31.4 ± 7.4	− 0.8 ± 4.6	0.409
Skeletal muscle mass (kg)	46.4 ± 12.1	46.1 ± 11.7	− 0.4 ± 2.0	0.382
AST (IU/L)	22.4 ± 6.7	20.2 ± 5.7	− 2.2 ± 6.6	0.106
ALT (IU/L)	25.3 ± 10.2	19.7 ± 6.6	− 5.6 ± 8.8	0.004*
γ-GTP (IU/L)	46.0 [25.0 – 61.0]	26.0 [19.0 – 50.0]	− 11.0 [− 25.0–1.0]	< 0.001*
Type IV collagen (ng/mL)	159.9 ± 81.5	157.7 ± 46.8	− 2.2 ± 39.8	0.785
FIB4 index	1.6 ± 0.8	1.7 ± 0.7	0.1 ± 0.4	0.795
FLI	68.0 ± 22.1	53.4 ± 27.7	− 14.6 ± 15.7	< 0.001*
UACR (mg/gCr)	158.8 ± 306.3	110.7 ± 255.6	− 48.1 ± 190.1	0.218
eGFR (mL/min/1.73m^2^)	68.3 ± 20.0	65.3 ± 21.0	− 3.0 ± 7.3	0.053
RBC (× 10^4^/μL)	440.8 ± 39.9	471.8 ± 38.8	31.0 ± 15.6	< 0.001*
Ht (%)	40.3 ± 3.5	43.4 ± 3.6	3.1 ± 1.8	< 0.001*
Plt (× 10^4^/μL)	21.6 ± 5.8	21.7 ± 5.6	0.1 ± 2.0	0.903
SBP (mmHg)	142.2 ± 14.9	131.9 ± 14.6	− 10.3 ± 14.4	0.002*
DBP (mmHg)	84.0 ± 9.3	81.4 ± 9.4	− 2.6 ± 8.5	0.145
CPR (ng/mL)	3.9 ± 1.9	2.9 ± 1.9	− 0.9 ± 2.2	0.044*

Data are presented as the mean ± standard deviation or median (interquartile range)

*Indicates statistical significance. Paired t-test, (versus 0 weeks) *p < 0.05, Wilcoxon signed-rank test, (versus 0 weeks) *p < 0.05

“Amount of change” is the change between 0 and 24 weeks

FPG: fasting plasma glucose; BMI: body mass index; AST: aspartate aminotransferase; ALT: alanine aminotransferase; γ-GTP: γ-glutamyl transpeptidase; FIB4 index: Fibrosis-4 index; FLI: fatty liver index; UACR: urinary albumin creatinine ratio; eGFR: estimated glomerular filtration rate; RBC: red blood cell count; Ht: hematocrit; Plt: platelet count; SBP: systolic blood pressure; DBP: diastolic blood pressure; CPR: serum C-peptide

### Multivariate analysis

Multivariate analysis was performed to identify the factors affecting the changes in items related to NAFLD, namely FLI, and ALT. The correlations of the changes in FLI and ALT levels with other parameters are summarized in Table 4. Among the items selected by stepwise analysis, changes in the TG level were most closely correlated to the FLI change. The item with the strongest correlation to the ALT level was the change in the AST level, thus indicating that body weight, HbA1c level, and lipid levels were not related to changes in liver enzyme levels. Further investigation using multiple regression analysis revealed that FLI was likely to decrease in patients with high baseline serum C-peptide levels. In addition, the analysis showed that ALT levels were likely to decrease in patients with high baseline body fat percentage, ALT, and serum C-peptide levels.

**Table 4:** Relationship of ΔFLI and ΔALT with changes in variables at 24 weeks (n = 25)

	ΔFLI		ΔALT	
	Correlation coefficient	p-value	Correlation coefficient	p-value
Δ HbA1c (%)	0.520	0.008*	0.598	0.002*
Δ Total cholesterol (mg/dL)	0.253	0.222	0.380	0.061
Δ TG (mg/dL)	0.727	< 0.001*	− 0.250	0.228
Δ HDL-C (mg/dL)	− 0.388	0.055	0.278	0.179
Δ LDL-C (mg/dL)	0.371	0.068	0.453	0.023*
Δ non-HDL-C (mg/dL)	0.396	0.050	0.288	0.162
Δ ApoB (mg/dL)	0.349	0.087	0.182	0.383
Δ ApoA-1 (mg/dL)	− 0.117	0.578	0.309	0.133
Δ TG/HDL-C ratio	0.695	< 0.001*	− 0.292	0.157
Δ RLP-C (mg/dL)	0.625	< 0.001*	0.144	0.492
Δ body weight (kg)	0.736	< 0.001*	0.427	0.033*
Δ BMI (kg/m^2^)	0.727	< 0.001*	0.320	0.119
Δ waist circumference (cm)	0.278	0.179	0.092	0.662
Δ body fat percentage (%)	0.462	0.020*	0.453	0.023*
Δ AST (IU/L)	0.278	0.179	0.835	< 0.001*
Δ ALT (IU/L)	0.493	0.012*	–	–
Δ γGTP (IU/L)	0.265	0.200	0.595	0.002*
Δ CPR (ng/mL)	0.722	< 0.001*	0.389	0.055
Δ FLI	–	–	0.493	0.012*

*Indicates statistical significance. Pearson’s test, (versus 0 weeks) *p < 0.05, Spearman’s test, (versus 0 weeks) *p < 0.05

Δ indicates the change between 0 and 24 weeks in each parameter

FLI: fatty liver index; HbA1c: glycated hemoglobin; TG: triglyceride; HDL-C: high-density lipoprotein-cholesterol; LDL-C: low-density lipoprotein-cholesterol; Apo: apolipoprotein; RLP-C: remnant-like lipoprotein cholesterol; BMI: body mass index; AST: aspartate aminotransferase; ALT: alanine aminotransferase; γ-GTP: γ-glutamyl transpeptidase; CPR: serum C-peptide

## Discussion

ApoA-1 is a major protein component of HDL-C. Both ApoA-1 and HDL-C suppress the progression of arteriosclerosis by binding to excess cholesterol in the periphery and transporting it to the liver [16]. However, it should be noted that high HDL-C levels do not necessarily reduce the risk of atherosclerosis. As for HDL, it is important to be able to extract excess cholesterol from the walls of the blood vessels, which is called the cholesterol efflux capacity [17]. A cholesteryl ester transfer protein inhibitor, Evacetrapib, which substantially increases the levels of HDL-C and lower LDL-C, reportedly did not reduce cardiovascular risk and mortality and hence is not being marketed [18]. Further, impaired glucose tolerance has been reported to reduce the ability of HDL to extract cholesterol [19]. HDL-C has two subspecies, namely HDL-C 2 and HDL-C 3. Large, cholesterol-rich HDL-C 2 is inversely associated with plasma TG and insulin resistance, while small, cholesterol-poor HDL-C 3 is not [20]. HDL-C subfractions or measurement of cholesterol efflux capacity was not performed in this study. Increased levels of ApoA-1 are more strongly associated with a lower risk of coronary artery disease, compared with increased levels of HDL-C [21]. Furthermore, ApoA-1 is associated with NAFLD. A Korean study reported that patients with lower ApoA-1 levels showed a higher prevalence of NAFLD, even if they were not obese [22]. In another study on mice, the incidence of NAFLD was higher in ApoA-1 deficient mice [23]. These reports suggest that lower levels of ApoA-1 are associated with a higher incidence of arteriosclerotic diseases and NAFLD.

ApoB is a protein component of LDL-C particles, and the ApoB level represents the number of LDL-C particles [24]. A higher ApoB level is associated with a higher risk of arteriosclerotic diseases and NAFLD [25, 26]. Similarly, RLP-C is a metabolite produced during the degradation of lipoproteins, such as very low-density lipoproteins (VLDLs) or chylomicrons. Higher RLP-C levels were associated with an increased incidence of arteriosclerotic diseases and NAFLD [27, 28] and a higher TG/HDL-C ratio with a higher risk of arteriosclerotic diseases and NAFLD [29, 30].

Our findings showed significant reductions in HbA1c and fasting plasma glucose levels, indicating a potential beneficial effect of luseogliflozin on diabetes control. Similar to other reports on luseogliflozin [9, 31], this study showed reductions in BMI, body weight, and waist circumference. Although the visceral fat mass was not measured accurately, waist circumference has been reported to correlate with the visceral fat mass area [32]. No reduction in skeletal muscle mass was observed in this study. Similarly in Sasaki’s study [9], there was only a small decrease in the skeletal muscle mass index, while BMI and the total fat mass decreased significantly. We routinely explained to the patients to continue the original diet and exercise therapy but did not ask them to follow a special diet or exercise intensively after starting luseogliflozin. Luseogliflozin is reported to regulate essential amino acids, short-chain fatty acids, and intestinal bacteria which leads to an increase in skeletal muscle mass [33]. Therefore, we believe that a significant reduction in skeletal muscle mass might not occur, however, it is important to consider individual differences and carefully evaluate the patients’ body compositions.

As for lipids, HDL-C and ApoA-1 levels significantly increased, indicating a reduced risk of arteriosclerotic diseases and NAFLD. Although we did not measure the cholesterol efflux capacity of HDL, it is reported that impaired glucose tolerance reduces cholesterol efflux capacity [19]. As such, an improvement in diabetes control, likely improves the cholesterol efflux capacity of HDL, leading to a reduced risk of arteriosclerotic diseases. Additionally, TG and RLP-C levels, as well as the TG/HDL-C ratio, significantly decreased, indicating a reduced risk of arteriosclerotic diseases and NAFLD. Therefore, changes in lipid levels can potentially reduce the risk and suppress the progression of diabetic complications. No significant changes were observed in LDL-C and ApoB levels. In a study by Ejiri et al. [34] using luseogliflozin in patients with diabetes and heart failure with baseline lipid profiles at normal range, no significant changes were observed in small dense LDL-C, TG, or LDL-C levels. In a retrospective analysis by Kubota et al. [35], 63 patients with T2DM on usual doses of SGLT2-i (Empagliflozin, Canagliflozin, Dapagliflozin, or Tofogliflozin), showed a significant decrease in total cholesterol and non-HDL-C levels, but no changes in TG, HDL, or LDL-C levels. However, this study was limited owing to its observational nature and use of multiple SGLT2-i. In another study of dapagliflozin, the LDL-C level showed no significant change. However, subsequent research revealed that the amount of small dense LDL-C, the main risk factor for arteriosclerosis among LDL particles, was sufficiently suppressed [36]. In our study, changes in the levels of small dense LDL-C could not be determined, as this assessment was not covered by insurance and therefore not measured. However, several surrogate markers, such as LDL-C/ApoB [37] and TG/ApoB [38] ratios, could indicate and identify the changes in small dense LDL-C. Hence, we evaluated these markers and compared them with those of the dapagliflozin study which showed a significant decrease in small dense LDL-C [36]. Surprisingly, the changes in these markers were similar to those in the dapagliflozin study. In our study, the LDL-C/ApoB ratio showed a change from 1.1 to 1.1 (p = 0.373), similar to that in the dapagliflozin study (change from 1.2 to 1.2, p value is not available). The TG/ApoB ratio showed a change from 2.4 to 1.9 (p = 0.007), similar to that in the dapagliflozin study (change from 1.5 to 1.3, p value is not available) (Additional file 3). Since the dapagliflozin study reported a significant reduction of small dense LDL-C, we recognized that the LDL-C/ApoB ratio is not accurate enough. On the other hand, the dapagliflozin study reported a significant reduction of the TG/ApoB ratio which was similar to our study that also showed a significant reduction of TG/ApoB, which likely meant reduction in the small dense LDL-C.

For assessing the liver function, FLI was measured to determine the risk of fatty liver, and the FIB-4 index and type IV collagen level were measured to screen for fibrosis in the liver. In our study, FLI was reduced significantly from 68.0 to 53.4 after 24 weeks (p < 0.01), whereas no significant changes were observed in fibrosis markers, which was similar to the findings of Sumida et al. at 24 weeks [31], however other studies with 52 weeks of follow-up showed a significant decrease [39, 40]. These differences could be due to the difference in the sample size and observation period. Changes in the FIB-4 index or type IV collagen level may be observed with even longer follow-ups or in patients with more severe liver dysfunction with fibrosis. Although the presence of fatty liver was not evaluated using images in this study, fatty liver was likely suppressed based on the FLI changes; indeed, a previous study reported a reduction in intrahepatic fat content following luseogliflozin administration in patients with T2DM and NAFLD [31]. Additionally, the ALT and γ-GTP levels were significantly decreased. A meta-analysis that aimed to evaluate the effect of SGLT2-i on NAFLD based on the changes in liver enzyme levels and liver fat volume concluded that SGLT2-i can significantly decrease ALT levels and liver fat, accompanied by weight loss [41]. Another study on Ipragliflozin in patients with diabetes and NAFLD also reported a significant decrease in the ALT and γ-GTP levels, along with the pathological improvement of NAFLD [42]; thus, luseogliflozin might reduce the risk of and improve NAFLD. Improvement in liver enzyme levels was observed, even though the study participants did not show severe liver dysfunction. Therefore, we deduced that earlier administration of SGLT2-i can suppress NAFLD progression over a period of 24 weeks.

While assessing the renal function, red blood cell count and hematocrit values increased significantly, which might not be due to dehydration, but due to the fibroblast-like cells regaining their ability to produce erythropoietin, because of the reduction in excessive glucose reabsorption in the renal tubules [43]. A decrease in the UACR was observed in this study, though statistically insignificant. Kubota et al. reported that UACR levels were significantly reduced only in the microalbuminuria and overt albuminuria groups, especially in patients with decreased body weight, blood pressure, and blood glucose [35]. As for luseogliflozin, a study reported a significant decrease in ACR [9], but two other studies did not, similar to our study [44, 45]. These differences could be probably due to variations in the sample size of the participants and the renal stages. Overall, luseogliflozin might have protective effects on the kidney and renal tubules.

Improvements in lipid profile and liver function may be attributed to many possible mechanisms. First, hyperglycemia and hyperinsulinemia cause fat accumulation in the liver, enlargement of adipose cells, and fat deposition in muscles, which could lead to insulin resistance [46]. When hyperglycemia and hyperinsulinemia are alleviated by SGLT2-i administration, these changes are alleviated, which eventually leads to the improvement of obesity and fatty liver. Our study also reports that indicators related to NAFLD, including FLI and ALT, are likely to decrease in patients with higher baseline serum C-peptide levels or body fat percentage, which means that patients with higher insulin resistance can benefit from SGLT2-i. Second, β-oxidation in the liver is also activated by SGLT2-i [4], leading to reduced production of VLDLs or RLP-C. Third, peroxisome proliferator-activated receptor alpha (PPAR-α), activated when SGLT2-i is administered [47], increases the production of ApoA-1, which eventually leads to the suppression and improvement of NAFLD [48].

Multivariate analysis in our study also revealed that the changes in the TG level were most closely correlated to the FLI change. Previous studies have shown that hypertriglyceridemia is one of the risk factors for NAFLD [49] and that the prevalence and degree of hypertriglyceridemia significantly correlate with the severity of NAFLD [50, 51]. It is known that the accumulation of triglycerides in hepatocytes increases fatty acid β-oxidation. When this occurs in the presence of mitochondrial abnormalities, it leads to a free radical formation with consequent cell injury, inflammation, and fibrosis [52]. Our study findings indicate that reducing TG levels is the key to improve liver function.

This study had several limitations. First, is the small sample size of 25 patients, and a shorter study period of 24 weeks. A larger and longer study is required to further investigate the durability of the changes. Second, the severity of liver dysfunction in the participants was mild, which should be taken into consideration; more changes might occur with a larger and longer-term evaluation, especially in patients with higher levels of liver enzymes. Changes in the measures for liver fibrosis, such as the FIB-4 index or type IV collagen, might need a longer follow-up of approximately 1 year. Third, levels of HDL-C, HDL-C subspecies (HDL-C 2 and HDL-C 3), or cholesterol efflux capacity were not measured in this study, which is required to precisely assess the anti-arteriosclerotic function of HDL. Fourth, we evaluated only the markers of fatty liver, but not the fatty liver itself. Even if the markers of fatty liver are improved, it is not clear whether the fatty liver is actually improved, as abdominal ultrasonography was not performed in this study. Furthermore, the most robust approach for diagnosing NAFLD needs the evaluation of TG accumulation via magnetic resonance imaging or vibration‐controlled transient elastography. Fifth, we did not perform a detailed lipoprotein analysis to clarify whether TG decline is mainly due to chylomicrons or VLDLs. TG-rich lipoproteins generally include VLDL and chylomicrons, but chylomicrons are less likely to cause arteriosclerosis unless they form remnants. A complete lipoprotein evaluation should be performed for further information. Sixth, the study did not measure the levels of small dense LDL-C as it was not covered by insurance; therefore, potential changes caused due to LDL-C remained undetected. Seventh, serum insulin levels were not measured. The measurement of serum insulin levels in patients who were not administered insulin might provide more information regarding the changes in insulin resistance. Lastly, although the dosage of drugs for diabetes or dyslipidemia did not change a month prior to or during the study, the potential effects caused by traditional treatments should be considered.

## Conclusions

Luseogliflozin-induced changes in the lipid profile were related to the suppression or improvement of arteriosclerotic diseases and liver function, respectively. Improvements in liver function and reductions in FLI were also observed, especially in patients with high baseline serum C-peptide levels. The improvement in TG level had the greatest effect on the improvement of FLI. SGLT2-i can also cause weight loss, which is important for NAFLD treatment. Luseogliflozin can lead to the suppression or improvement of diabetes, obesity, and diabetic complications. The study suggests that earlier use of SGLT2-i might be beneficial in preventing diabetic complications.

## Supplementary Information

**Additional file 1: Figure S1**. Overview of the study. This figure provides an overview of the 25 outpatients with T2DM who were enrolled at Minami Osaka Hospital.

**Additional file 2: Table S1**. Study inclusion and exclusion criteria. This table lists the study inclusion and exclusion criteria.

**Additional file 3: Table S2**. Change in the LDL-C/ApoB and TG/ApoB ratios (n = 25). This table shows the changes in LDL-C/ApoB and TG/ApoB ratios from 0 to 24 weeks.

## Data Availability

All data generated or analyzed during this study are included in this published article.

## Abbreviations

ALT: Alanine aminotransferase
Apo: Apolipoprotein
AST: Aspartate aminotransferase
BMI: Body mass index
eGFR: Estimated glomerular filtration rate
FIB4 index: Fibrosis-4 index
FLI: Fatty liver index
HbA1c: Glycated hemoglobin
HDL-C: High-density lipoprotein-cholesterol
LDL-C: Low-density lipoprotein-cholesterol
NAFLD: Non-alcoholic fatty liver disease
PPAR-α: Peroxisome proliferator-activated receptor alpha
RLP-C: Remnant-like lipoprotein cholesterol
SGLT2-i: Sodium-glucose transporter 2 inhibitors
T2DM: Type 2 diabetes mellitus
TG: Triglyceride
UACR: Urinary albumin-creatinine ratio
VLDL: Very low density lipoprotein
γ-GTP: γ-Glutamyl transpeptidase
