# Supplementary material for: Beneficial effects of luseogliflozin on lipid profile and liver function in patients with type 2 diabetes mellitus (BLUE trial): a single-center, single-arm, open-label prospective study
Source: Diabetol Metab Syndr. 2023 May 11;15:97. doi: 10.1186/s13098-023-01074-1 (PMC10173585; doi:10.1186/s13098-023-01074-1)
Supplement: Supplementary file 2 — Additional file 2: Table S1. Study inclusion and exclusion criteria. This table lists the study inclusion and exclusion criteria. [file 13098_2023_1074_MOESM2_ESM.docx]

**Additional File 2: Table S1**. Inclusion and exclusion criteria

| **Inclusion criteria** |
| --- |
| 1) Patients with T2DM who received diet, exercise, and glucose-lowering therapy for at least a month before participation and had HbA1c levels of ≥7.0% and ≤10.0%  2) Patients with fasting plasma TG ≥ 120 mg/dL and ≤ 400 mg/dL  3) Patients with an eGFR of at least 30 mL/min/1.73 m^2^  4) Patients 20 years or older  5) Patients who agreed to participate in this study  6) Patients with a BMI of 20 kg/m^2^ or higher |
| **Exclusion criteria** |
| 1) Patients with T1DM  2) Patients who had a change in their dose or usage of antidiabetic or dyslipidemia drugs within the previous month  3) Patients who used SGLT2-i within the previous month  (Patients who suspended their use of SGLT2-i for more than a month were eligible to participate)  4) Patients with a history of severe ketosis, diabetic coma, or pre-coma within 6 months before participation  5) Patients with proliferative retinopathy  6) Patients with eGFR <30 mL/min/1.73 m^2^ at screening, or patients who needed renal dialysis  7) Patients with dysuria  8) Patients with urinary tract infections or genital infections  9) Patients who have a severe infection or serious injury  10) Patients who were operated on or planned for an operation during the study period  11) Patients with a history of cerebrovascular attack, unstable angina, myocardial infarction, angioplasty, or severe heart disease (NYHA Classification III-IV) within 6 months  12) Patients who were pregnant, breastfeeding, or planning to become pregnant  13) Patients with severe liver dysfunction (Child-Pugh score: ≥ 10 and ≤ 15)  14) Patients with a history of allergy to luseogliflozin  15) Patients who consumed alcohol (more than 20 g/day for women and 30 g/day for men)  16) Patients evaluated by an investigator to be inappropriate as a study participant |

T2DM, type 2 diabetes mellitus; HbA1c, glycated hemoglobin; TG, triglycerides; eGFR, estimated glomerular filtration rate; BMI, body mass index; T1DM, type 1 diabetes mellitus; SGLT2-i, sodium-glucose transporter 2 inhibitor; NYHA, New York Heart Association
