# Supplementary material for: Beneficial effects of luseogliflozin on lipid profile and liver function in patients with type 2 diabetes mellitus (BLUE trial): a single-center, single-arm, open-label prospective study
Source: Diabetol Metab Syndr. 2023 May 11;15:97. doi: 10.1186/s13098-023-01074-1 (PMC10173585; doi:10.1186/s13098-023-01074-1)
Supplement: Supplementary file 3 — Additional file 3: Table S2. Change in the LDL-C/ApoB and TG/ApoB ratios (n = 25). This table shows the changes in LDL-C/ApoB and TG/ApoB ratios from 0 to 24 weeks. [file 13098_2023_1074_MOESM3_ESM.docx]

**Additional File 3: Table S2**. Change in the LDL-C/ApoB and TG/ApoB ratios (n = 25)

|  | 0 weeks | | |  | 24 weeks | | |  | Amount of change | | |  | p-value |
| --- | --- | --- | --- | --- | --- | --- | --- | --- | --- | --- | --- | --- | --- |
| LDL-C/ApoB | 1.1 | ± | 0.2 |  | 1.1 | ± | 0.2 |  | 0.0 | ± | 0.1 |  | 0.373 |
| TG/ApoB | 2.4  [1.8 – 2.6] | | |  | 1.9  [1.2 – 2.1] | | |  | -0.5  [-1.0 – -0.1] | | |  | 0.007* |

Data are presented as the mean ± standard deviation or median (interquartile range).

*Indicates statistical significance. Paired t-test, (versus 0 weeks) *p < 0.05; Wilcoxon signed-rank test, (versus 0 weeks) *p < 0.05.

“Amount of change” is the change between 0 and 24 weeks.

LDL-C, low-density lipoprotein-cholesterol; Apo, apolipoprotein; TG, triglyceride.
